# Disseminated cysticercosis and Kaposi sarcoma in a child with HIV/AIDS: A case report

**DOI:** 10.1186/s12879-020-05039-x

**Published:** 2020-04-25

**Authors:** David W. McCormick, Jason M. Bacha, Nader K. El-Mallawany, Carrie L. Kovarik, J. S. Slone, Liane R. Campbell

**Affiliations:** 1 Baylor College of Medicine, 1 Baylor Plaza BCM 620, Houston, TX 77030-3411 USA; 2 Baylor International Pediatric AIDS Initiative (BIPAI) at Texas Children’s Hospital, Baylor College of Medicine, Houston, TX USA; 3 Baylor College of Medicine Children’s Foundation - Tanzania, Pediatrics, Mbeya, Tanzania; 4 Baylor College of Medicine - Texas Children’s Cancer and Hematology Centers, Houston, TX USA; 5 Department of Dermatology, Perelman School of Medicine at the University of Pennsylvania, Philadelphia, PA USA

**Keywords:** Kaposi sarcoma, Cysticercosis, *Taenia solium*, HIV, AIDS, Opportunistic infection

## Abstract

**Background:**

Clinical manifestations of extraneural infection with the pork tapeworm *Taenia solium* typically affect the muscles, eyes, alimentary canal, and/or subcutaneous tissues. Children living with HIV are at increased risk for more widespread and severe manifestations of food-borne opportunistic infections, including *T. solium*, due to fluctuating levels of immunosuppression. We present a case of disseminated *T. solium* in a HIV-positive child with Kaposi sarcoma living in Tanzania with cysticercosis presenting as widespread subcutaneous nodules.

**Case presentation:**

A 4-year-old HIV-positive boy in Southern Tanzania presented for evaluation of > 30 violaceous skin lesions, few subcutaneous nodules, and a circumferential violaceous penile lesion which rapidly grew after initiation of ART. The patient was clinically diagnosed with Kaposi sarcoma and started on chemotherapy with bleomycin, vincristine, and doxorubicin. He completed 10 cycles of chemotherapy, with full resolution of the violaceous skin and penile lesions but persistence of his subcutaneous nodules, thus paclitaxel was added. After 12 additional cycles of paclitaxel, his subcutaneous nodules enlarged, and biopsy of a scapular subcutaneous nodule was performed. Histopathology revealed a cystic structure with a central larval scolex and serrated spiral canal consistent with *T. solium,* which confirmed a diagnosis of disseminated cysticercosis. He completed a 10-day course of praziquantel and albendazole with resolution of the subcutaneous nodules.

**Conclusions:**

Disseminated cysticercosis is an unusual opportunistic infection which can present as subcutaneous nodules without other typical cysticercosis symptoms. Immunosuppression – from HIV and/or chemotherapy – may unmask cysticercosis in children in endemic regions and result in more severe manifestations of this disease. Cysticercosis should remain on a clinician’s differential for subcutaneous nodules, especially in children living with HIV. Cysticercosis can mimic Kaposi sarcoma, and histopathology is essential to accurately diagnose and manage patients with concerning skin lesions.

## Background

Cysticercosis is an infection with the larval form of *Taenia solium*, the pork tapeworm. Cysticercosis is transmitted through fecal contamination of food or water or autoinfection, and persons with taeniasis (the intestinal form) serve as the reservoir. Poor food preparation and hand hygiene increases the risk of cysticercosis [1]. While mild or asymptomatic intestinal infection commonly occurs following ingestion of contaminated pork, there are two important, more severe clinical manifestations of cysticercosis that can occur following ingestion of food or water contaminated with cysts, depending on the site of larval encystation*.* Neurocysticercosis (NCC) refers to encystation of cysticerci within the brain parenchyma or subarachnoid space, and is the leading cause of seizures in low- and middle-income countries (LMIC) [2]. Disseminated cysticercosis (DC) refers to cysticerci migrating to and encystation in skeletal muscle, cardiac muscle, eyes, and/or subcutaneous tissues.

Cysticercosis is one of the most prevalent helminth infections globally, with an estimated 370,000 persons affected by NCC [3]. Data specific to sub-Saharan Africa (SSA) are limited; however, regional SSA data suggest a higher prevalence than in South America and the Indian subcontinent [4], and a major cause of disability-adjusted life-years in SSA [3]. In Tanzania, recent estimates suggest that approximately 16% of the population have DC or NCC, and 2–5% of the population has taeniasis [5].

Disseminated cysticercosis is an uncommon manifestation of infection with *T. solium* and most commonly presents as subcutaneous and intramuscular nodules, although any organ can be infected [6–10]. Among people living with HIV, a recent case-control study did not find any association between HIV infection and NCC in Tanzania [11], and to date, no unique relationship between NCC and HIV has been reported [12]. We present a case of disseminated cysticercosis in a HIV positive child being treated for Kaposi sarcoma in Tanzania that presented as widespread subcutaneous lesions following initiation of antiretroviral therapy (ART) and chemotherapy.

## Case presentation

**Fig. 1:**
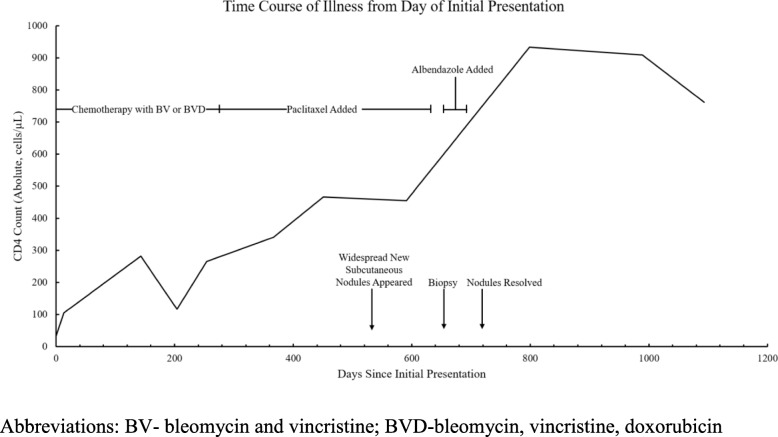
Timeline of clinical course from day of presentation showing absolute CD4 count and major clinical events

**Fig. 2:**
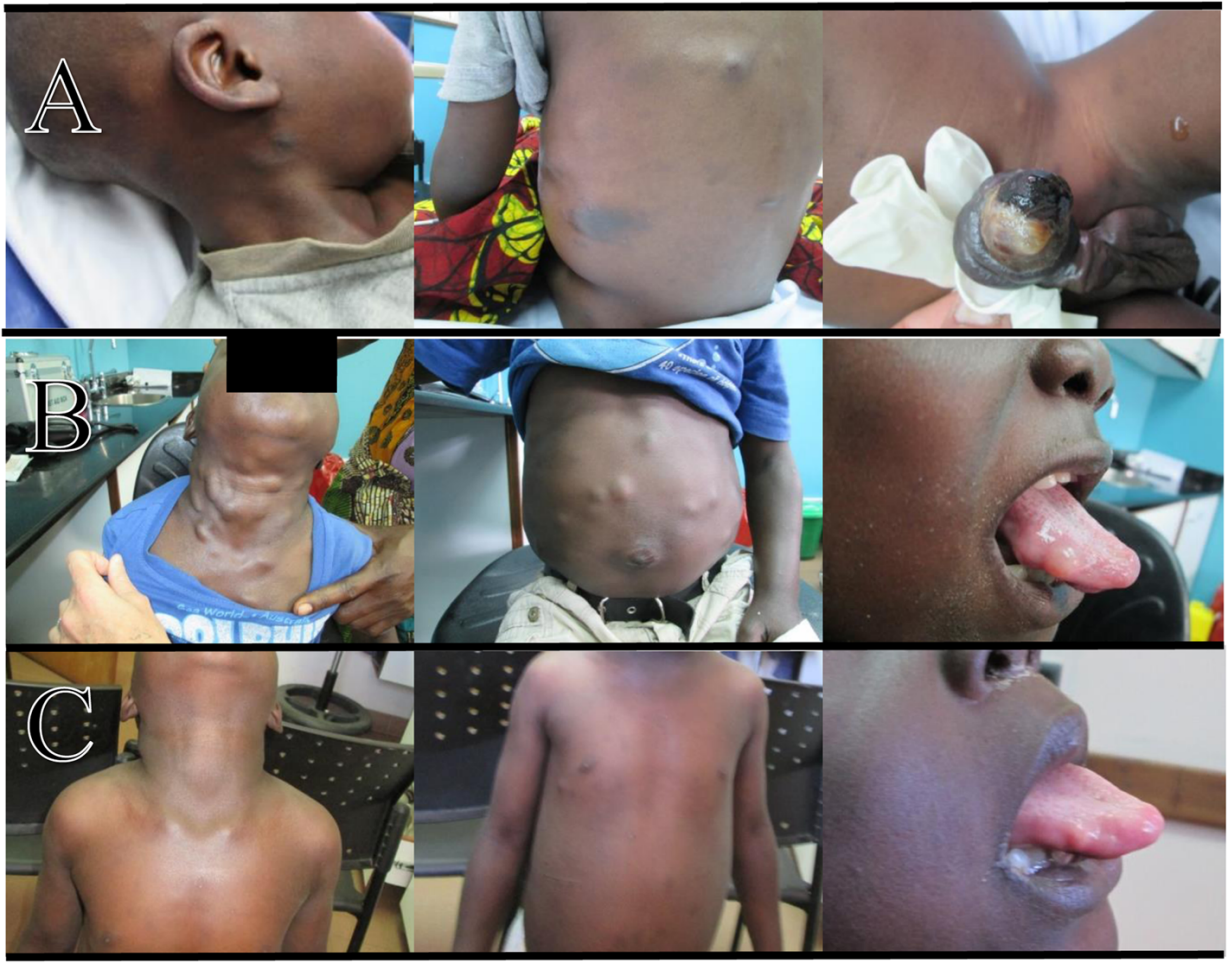
Clinical images showing lesions (**a**) at the time of diagnosis of KS-IRIS, (**b**) following antineoplastic therapy, and (**c**) following antihelminthic therapy

An HIV-positive 4-year-old boy presented to clinic for evaluation of worsening skin lesions after starting ART. His absolute (percentage) CD4 count at the time of ART initiation was 32 cells/μL (3%) (Fig. 1), and he was started on ART with zidovudine-lamivudine-nevirapine. Approximately 3 months after ART initiation, he developed > 30 violaceous papules and plaques, a necrotic edematous circumferential plaque on the distal shaft and glans of the penis, and scattered small subcutaneous nodules in his submandibular and cervical regions (Fig. 2, panel a). He was clinically diagnosed with Kaposi sarcoma secondary to the immune reconstitution inflammatory syndrome due to the classic appearance of his cutaneous lesions. His ART was shifted to abacavir-lamivudine-lopinavir/ritonavir due to anemia (hemoglobin 7.2 g/dL) and potential activity of protease inhibitors against human herpes virus-8 [13, 14]. He was started on a chemotherapy regimen of bleomcyin (15 U/m^2^) and vincristine (1.4 mg/m^2^) every 2 weeks. Biopsy was not obtained initially due to logistical and resource constraints.

After five cycles of this chemotherapeutic regimen, doxorubicin (25–35 mg/m^2^) was added based on failure to achieve complete clinical remission. The patient completed an additional 10 cycles of bleomycin-vincristine-doxorubicin every 3 weeks without complete remission of the violaceous lesions or penile nodule. During the course of chemotherapy, he achieved full virologic suppression and his CD4 count had increased to 265 cells/μL (13%) after 143 days of antiretroviral therapy (Fig. 1). However, his KS lesions continued to persist, and chemotherapy was shifted to monthly paclitaxel. He eventually completed 12 cycles of paclitaxel monotherapy with dexamethasone as a premedication and showed improvement and full resolution of the violaceous skin and penile lesion. However, during this time he developed numerous additional subcutaneous nodules on his trunk, tongue, and extremities that rapidly and progressively increased in size in number (Fig. 2, panel b).

**Fig. 3:**
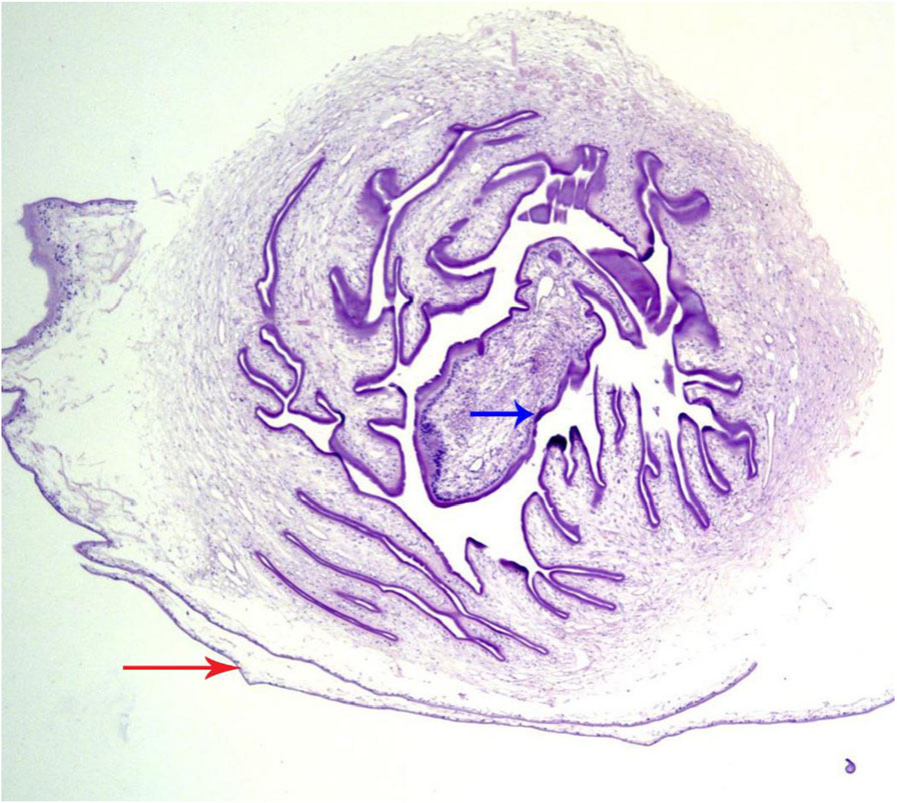
Biopsy of the patient's subcutaneous nodule (4x magnification, hemtoxylin and eosin stain) showing a *Taenia solium* cysticercus. Cysticerci are fluid-filled cystic structures consisting of a thin bladder wall (red arrow) and a parenchymatous portion containing a scolex surrounded by a convoluted spiral canal (blue arrow). The hooks of the scolex are often only seen in few tissue sections

A biopsy was taken for further investigation. Histopathology of the nodule revealed a cystic structure with a central larval scolex and serrated spiral canal consistent with *T. solium*, which confirmed the diagnosis of DC (Fig. 3). Additional history taking with the caregiver revealed that the patient and his family consumed pork frequently as a regular part of their diet.

Chemotherapy was stopped and he was treated for DC with albendazole 7.5 mg/kg twice daily and praziquantel 25 mg/kg twice daily for 10 days. Prednisone was administered as an adjunctive treatment to limit the possibility of cerebral edema as NCC could not be ruled out (computed tomography scans of the head were unavailable). He had almost complete resolution of all subcutaneous nodules during his 10-day treatment course (Fig. 2, panel c). All treatment was well-tolerated with minor toxicity (anemia). A small sublingual nodule persisted acutely following treatment, fully resolving several months later. He has not had recurrence of KS or DC after 42 months of follow-up.

## Discussion and conclusions

Given the limited diagnostic capabilities, it is difficult to determine if the DC in this patient was a complication of primary infection with *T. solium*, or secondary to underlying immunodeficiency secondary to HIV/AIDS, chemotherapy, steroid administration, or reconstitution of the immune system following ART initiation. Infection with HIV has not been associated with increased prevalence of cysticercosis or increased severity of disease; however, prevalence of helminth infections seems to peak with a CD4 count of 200–500 [11, 12, 15].There is no clear association between HIV infection and cysticercosis [11, 12], and it is difficult to determine if the subcutaneous nodules seen in our patient represent primary infection or unmasking of extant lesions following reconstitution of the immune system.

The presentation of DC following ART and chemotherapy is a reminder that clinicians need to consider DC in the differential for CLHIV living in areas with high burden of cysticercosis and/or other helminthic disease who develop widespread subcutaneous nodules during treatment. The differential diagnosis for cutaneous cestode infections in children in SSA includes sparganosis and echinococcosis, which should be also be considered when an immunocompromised person’s clinical course worsens or fails to improve [16, 17]. Clinicians in endemic regions must be familiar with the signs and symptoms of all forms of *T. solium* infection in CLHIV, which include taeniasis, subcutaneous nodules, NCC, or otherwise unexplained nodules or lesions in muscle, organs, or other soft tissue. Biopsy of lesions not responding as expected to treatment is recommended to make a definitive diagnosis and identify the most appropriate treatment.

## Data Availability

The data used and/or analysed during this case report are available from the corresponding author on reasonable request.

## Abbreviations

ART: Antiretroviral therapy
AIDS: Acquired Immunodeficiency Syndrome
CD4: Cluster of Differentiation 4 cell
CLHIV: Children living with HIV
DC: Disseminated cysticercosis
KS: Kaposi Sarcoma
HIV: Human Immunodeficiency Virus
LMIC: Low- and middle-income countries
NCC: Neurocysticertosis
SSA: Sub-Saharan Africa
